# Peripheral Neuropathic Pain: From Experimental Models to Potential Therapeutic Targets in Dorsal Root Ganglion Neurons

**DOI:** 10.3390/cells9122725

**Published:** 2020-12-21

**Authors:** Ti-Yen Yeh, I-Wei Luo, Yu-Lin Hsieh, To-Jung Tseng, Hao Chiang, Sung-Tsang Hsieh

**Affiliations:** 1 Department of Anatomy and Cell Biology, College of Medicine, National Taiwan University, Taipei 10051, Taiwan; tiyenyeh@ntu.edu.tw; 2 Department of Life Science, College of Life Science, National Taiwan University, Taipei 10617, Taiwan; mzwesley@gmail.com; 3 Department of Anatomy, School of Medicine, College of Medicine, Kaohsiung Medical University, Kaohsiung 80708, Taiwan; yulinhsieh@icloud.com; 4 School of Post-Baccalaureate Medicine, College of Medicine, Kaohsiung Medical University, Kaohsiung 80708, Taiwan; 5 Department of Medical Research, Kaohsiung Medical University Hostpital, Kaohsiung 80708, Taiwan; 6 Department of Anatomy, School of Medicine, Chung Shan Medical University, Taichung 40201, Taiwan; tjtseng@csmu.edu.tw; 7 Department of Medical Education, Chung Shan Medical University Hospital, Taichung 40201, Taiwan; 8 Akouos, Inc., Boston, MA 02210, USA; rhchiangntu@gmail.com; 9 Department of Neurology, National Taiwan University Hospital, Taipei 10002, Taiwan; 10 Graduate Institute of Brian and Mind Sciences, College of Medicine, National Taiwan University, Taipei 10051, Taiwan; 11 Center of Precision Medicine, College of Medicine, National Taiwan University, Taipei 10055, Taiwan

**Keywords:** neuropathic pain, peripheral sensitization, dorsal root ganglia, transcriptional regulation, post-translational modification

## Abstract

Neuropathic pain exerts a global burden caused by the lesions in the somatosensory nerve system, including the central and peripheral nervous systems. The mechanisms of nerve injury-induced neuropathic pain involve multiple mechanisms, various signaling pathways, and molecules. Currently, poor efficacy is the major limitation of medications for treating neuropathic pain. Thus, understanding the detailed molecular mechanisms should shed light on the development of new therapeutic strategies for neuropathic pain. Several well-established in vivo pain models were used to investigate the detail mechanisms of peripheral neuropathic pain. Molecular mediators of pain are regulated differentially in various forms of neuropathic pain models; these regulators include purinergic receptors, transient receptor potential receptor channels, and voltage-gated sodium and calcium channels. Meanwhile, post-translational modification and transcriptional regulation are also altered in these pain models and have been reported to mediate several pain related molecules. In this review, we focus on molecular mechanisms and mediators of neuropathic pain with their corresponding transcriptional regulation and post-translational modification underlying peripheral sensitization in the dorsal root ganglia. Taken together, these molecular mediators and their modification and regulations provide excellent targets for neuropathic pain treatment.

## 1. Introduction

### 1.1. Overview of Neuropathic Pain

The prevalence of chronic pain with neuropathic characteristics has been reported to be over 7% of the general population in the world but this number could be underestimated because of the difficulty in characterization and poorly understood mechanisms [1]. The International Association for the Study of Pain (IASP) indicated that health-related quality of life is low in people suffering from neuropathic pain. Although scientists have been working hard to investigate relevant signal transductions in neuropathic pain for decades, only <50% patients could have a relief from neuropathic pain after medications due to the complicated signal transductions playing in neuropathic pain pathways and circuits [2,3]. The lesions in the central or peripheral nervous system reduce the threshold and increase the responses to the nociceptive stimulation. In peripheral nerve injury, the nociception is conveyed by the primary sensory neurons in the dorsal root ganglia (DRG) and back to secondary sensory neurons.

Nerve fibers are classified into different types including A, B, and C fibers according to their diameter, myelination status, and conduction velocity. In addition to the lesions in the central nervous system causing neuropathic pain, peripheral nerve degeneration (peripheral neuropathy) also induces neuropathic pain by affecting small myelinated Aδ fibers and unmyelinated C fibers [4]. The small myelinated Aδ fibers and unmyelinated C nerve fibers are mainly affected in peripheral neuropathic pain syndromes. Clinical symptoms of peripheral neuropathy include tingling, burning, and numbness due to dysregulation of nerve fibers [5]. Till now, although antidepressants, anticonvulsants, and opioids were recommended in the guidelines for treating neuropathic pain, the responses of treatments are not satisfactory and large scale of studies are continuously conducted to explore the whole pictures of neuropathic pain in different fields.

To investigate the detailed mechanisms of neuropathic pain, well-established in vivo models have been applied to mimic different etiologies of neuropathic pain such as the injury-induced and systemic exposure to neurotoxins. In spite of the fact that the proportion of peripheral sensitization driven neuropathic pain is difficult to distinguish, focusing on peripheral desensitization could be a phased strategy for treating peripheral neuropathic pain before intervene into central sensitization. Compared to the central nerve system, the permeability in DRG is relatively higher due to the incomplete blood–nerve barrier [6,7]. Neurotoxins could easily penetrate the blood–nerve barrier into the DRG and cause neuronal excitability [8]. The level of molecular expression in DRG neurons at the levels of gene and protein vary in neuropathic pain [9,10,11]. Converging researches showed that manipulation of DRG could relieve pain through changing the excitability and inhibiting signaling pathways [12,13,14,15,16,17]. Furthermore, the therapeutic region could be focal and easily restricted by targeting specific levels of DRG. Therefore, the molecules in DRG could be potential therapeutic targets in neuropathic pain. In this review, we will summarize the pain related molecules in neuropathic pain and focus on the peripheral sensitization within post-translational control and transcriptional modifications in DRG.

### 1.2. Etiology and Patholoigcal Approches

The challenge in managing neuropathic pain is to identify the exact etiology for appropriate treatment due to different etiologies affect various types of never fibers [18]. Both large and small fibers in the peripheral nervous system are affected in injury-induced neuropathic pain models. However, some disease such as chemotherapy neuropathy, vasculitic neuropathy, and Charcot–Marie–Tooth disease mainly target large diameter nerve fibers. Resiniferatoxin (RTX), one of the neurotoxins which belongs to capsaicin analogues that induces the desensitization in nociceptive neuron. However, RTX only affects small diameter nerve fibers specifically. Intraepidermal nerve fiber (IENF) density from the skin biopsy with immunohistochemistry staining of protein gene product 9.5 could be a standard and useful tool to estimate the degeneration of small-diameter sensory neurons in these neuropathic pain models [19]. Whereas, sural sensory nerve action potential (SNAP) and sural nerve morphometry could be performed to examine the pathological finding in larger fiber neuropathy [20].

## 2. Neuropathic Pain Models

### 2.1. Injury Induced

Several surgical neuropathic pain models have been developed decades and are widely used to mimic injury-induced neuropathic pain, including spinal nerve ligation (SNL), chronic constriction injury (CCI), partial sciatic nerve injury, and spared nerve injury (SNI) [21,22,23]. Each model has stable, long-lasting, and reproducible characteristics of neuropathic pain behavior, such as mechanical allodynia and thermal hyperalgesia but with corresponding unique pain behavior patterns [24]. The major factor distinguishing various injury-induced neuropathic pain models resides in the location of manipulations leading to similarities and differences (Table 1). We first will focus on the application of these injury induced neuropathic pain models.

Decompression is usually performed to relief the compression-induced neuropathic pain clinically. Although the variations exist in each operation, CCI produces spontaneous pain characteristic with four loose ligatures on the sciatic nerve. We performed the decompression at postoperative week four by removing the ligatures to investigate the mechanisms and temporal course of neuropathic pain [25]. Plasticity in the dorsal horn and partial reinnervation of the skin contributed to the normalization of neuropathic pain behavior [25,26].

Compared to CCI, the surgical procedure of SNL is much more complex and time consuming [24]. However, the variation in SNL is much lower given that same degree of injured spinal nerves in each operation. SNL separates the injured and uninjured DRG by ligating spinal nerve at the specific level and provides an opportunity to inject chemicals or gene constructs through the spinal nerve [27]. By combining spinal nerve injection and SNL model, the efficiency of therapeutic agents on injured DRG can be easily addressed.

### 2.2. Systemic Exposure Neuropathic Pain Model

In about 50% of sensory neuropathy, small fiber is predominantly affected [28]. Various diseases affect specific type of nerve fibers. For example, taxanes and vincristine mainly affect large fiber, whereas small fibers are affected at the early stage of diabetes mellitus [29,30]. The surgical neuropathic pain models that are mentioned above usually damage the nerve fiber without fiber type preference. Therefore, to establish suitable neuropathic pain model that mimics small or large fiber neuropathy is necessary for studying the disease like chemotherapy-induced neuropathy and painful diabetic peripheral neuropathy.

#### 2.2.1. Resiniferatoxin

**Figure 1:**
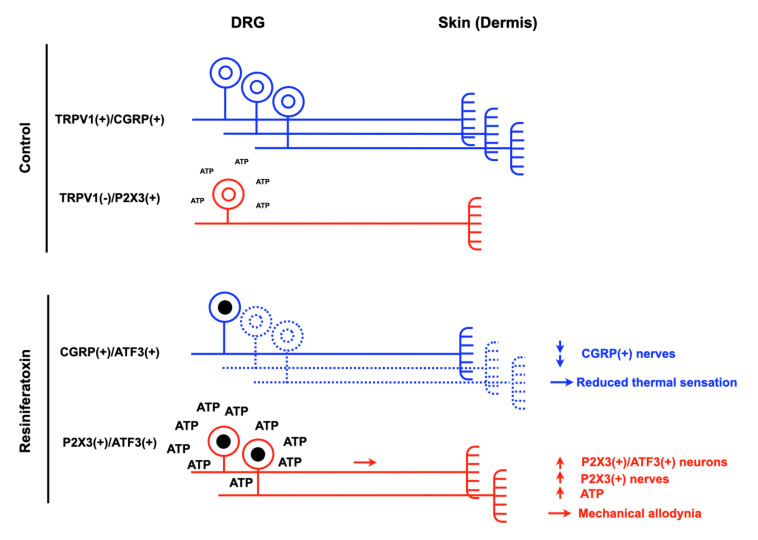
Schematic diagram illustrating the peripheral sensitization in resiniferatoxin (RTX)-induced neuropathic pain model. Thermal hypoalgesia and mechanical allodynia were observed after 1 week of RTX treatment. After RTX exposure, several dorsal root ganglia (DRG) profiles were changed including the increase of purinergic receptor P2X3 (P2X3)(+)/activating transcription factor 3 (ATF3)(+) neurons and the decrease of transient receptor potential vanilloid subtype 1 (TRPV1)(+)/calcitonin gene-related peptide (CGRP)(+) neurons. At the terminal ending of nerve, the phenotype of dermal nerve also had similar changes as the DRG. The number of CGRP(+) and P2X3(+) nerve were decreased and increased respectively.

Resiniferatoxin, an analog of capsaicin, is widely used for generating a small fiber neuropathy in rodents [31]. Hsieh et al. established a small fiber neuropathic pain model by using a single dosage of RTX. Experimental mice developed a pure small fiber neuropathy one week after an exposure to 50 μg/kg of RTX through intraperitoneal injection [32]. The number of injury marker (activating transcription factors 3 (ATF3)) was significantly increased only in small DRG neurons and the large DRG neurons remained ATF3 (−). Reduced of IENF density, mechanical allodynia, and thermal hypoalgesia were the characteristic pathology and behaviors in this model (Figure 1). To investigate the strategy for small fiber reinnervation in RTX-induced small fiber neuropathy, 4-methylcatechol (4-MC) could be used as a positive control for screening therapeutic candidate chemicals [31,33].

#### 2.2.2. Acrylamide

Acrylamide is one of the neurotoxins that targets the central and peripheral nervous system [34]. Both large and small nerve fibers could be affected by acrylamide through different exposure routes [35,36]. The unique pathology of acrylamide-induced neuropathy (“dying-back” phenomena) indicated the early degeneration of axons at the distal end. Each stage of neuropathy induced by acrylamide is distinct and could be classified by the neurological symptoms [36]. With accumulated acrylamide (35–140 mg/kg) by appropriate low-dose and repeated exposure, experimental animals showed mechanical allodynia and thermal hyperalgesia but without motor deficits [37]. Induction of massive oxidative stress is one of the neurotoxic mechanisms [38]. Treating antioxidants could reduce the accumulation of reactive oxygen species (ROS) in in vitro cell models and reverse the neurotoxicity that induced by acrylamide in in vivo animal model [39,40].

#### 2.2.3. Chemotherapy-Induced Peripheral Neuropathy (CIPN)

Various adverse effects occur when patients receive chemotherapy. About 60% of patients on chemotherapy in one month develop a chemotherapy-induced peripheral neuropathy with the “glove and stocking” type distribution [41]. The symptoms of CIPN sometimes might continue for several months even after the chemotherapy is completed. Different chemotherapy drugs target different types of nerve fibers [42]. The platinum-based drugs affect the large fibers and vincristine affects the nerve fibers without fiber type preference. Not only the type of fibers but also the target site in the neurons could be used to distinguish these chemotherapy drugs. Taxanes and vincristine target axons, whereas platinum-based drugs (cisplatin and oxaliplatin) target the soma of DRG [43,44,45]. The blood–nerve barrier in DRG is relatively permeable which leads to the penetrating of chemotherapeutic agents from the blood vessel into DRG [6]. Axonal demyelination and degeneration accompanied with mitochondrial dysfunction, axon transport inhibition, and calcium homeostasis are the major pathological findings in CIPN [46]. To date, there are no effective strategies to prevent CIPN during chemotherapy. Therefore, to establish a neuropathic pain model mimic CIPN is important for investigating the detailed mechanisms of CIPN.

The cumulative doses of these chemotherapy drugs (paclitaxel: 4 mg/kg, cisplatin: 23 mg/kg, and vincristine: 200 μg/kg) induced CIPN in animals [47]. Since the demyelinated large fibers are observed in CIPN, a semi-thin section for nerve morphometric analysis could be used for monitoring the status of demyelination in large fiber by calculating the number and the diameter of myelinated and demyelinated nerve fibers [48]. Combining CIPN models with electrophysiological examinations and nerve morphometry, Chine et al. observed that the expression of a regeneration-related protein, heat shock protein 27, in neurons which could reverse the symptoms of paclitaxel-induced CIPN by inhibiting the apoptosis, demyelination, and mitochondria swelling [49].

## 3. Molecular Targets in Dorsal Root Ganglia

Numerous molecules and pathways have been indicated as potential biomarkers or regulators in neuropathic pain [50]. This review aims to focus on the differences in the expression levels of well-established mediators in the DRG. These renowned mediators include P2X receptor family, the transient receptor potential (TRP) channels, voltage-gated sodium channels (NaVs), and voltage-gated calcium channels (CaVs) (Table 2 and Table 3).

### 3.1. Purinergic P2X Receptors (P2Xs)

The P2X family receptors belong to cation-permeable ionotropic receptor that can be activated by ATP. P2X receptors are expressed in the spinal cord, dorsal horn, and DRG neurons [51,52,53]. Accumulating evidence has indicated that P2X receptors, in particular, homomeric purinergic receptor P2X3 (P2X3) and heteromeric P2X2/3 receptors, are involved in spinal nociceptive transmission [54]. Thus, P2X2/3 have been proposed as potential therapeutic targets for peripheral neuropathic pain.

The expression level of P2X receptors is variable in different models of neuropathic pain. Rats with SNL had upregulated, downregulated, or unchanged levels of P2X3 expression in DRG [55,56,57]. Nevertheless, researchers have demonstrated that P2X3 exhibits an upregulation in the small to medium size neurons of lumbar DRGs in CCI-treated rats [51,52,53,58]. In the SNI model, the expression level of P2X3 was unchanged in rats. The reason behinds these differences in neuropathic pain models have not yet been fully understood. Additional investigations are required to investigate the roles of P2X receptors in injury-induced neuropathic pain models.

Hsieh et al. had shown that upon RTX exposure, peripherin (+) neurons were reduced and P2X3 (+) neurons were increased [59]. The upregulation of P2X3 (+) neurons was correlated with mechanical allodynia. These findings suggest that P2X3 is responsible for peripheral sensitization of neuropathic pain. Hori et al. found that cisplatin injection upregulated P2X3 expression [60]. This study provided findings of the involvement of P2X3 receptor in neuropathic pain.

#### 3.1.1. Transient Receptor Potential Receptors

Transient receptor potential receptors are non-selective channels that can induce painful sensations by detecting physical and chemical stimuli in the environment. In the primary afferent neurons of DRG, these receptors act as transducers for various kinds of stimuli including mechanical, thermal, and chemical stimuli. TRP receptors are classified into different subfamilies, and each subfamily is slightly different, resulting in detecting the diverse sensory stimuli. Of all of the subfamilies, the transient receptor potential vanilloid (TRPV), transient receptor potential ankyrin (TRPA), and transient receptor potential melastatin (TRPM) contribute to neuropathic pain [61]. In addition to being activated by their respective agonists, different TRP receptors respond to their unique temperature thresholds. For example, TRPV subtype 1 (TRPV1) channels are activated by elevating the temperature above 43 °C. Due to this special property, TRPV1 has been associated with thermal hyperalgesia in animal models [62,63]. The TRPA subtype 1 (TRPA1) channel, on the other hand, has a temperature threshold of 18 °C or under and thus is associated with cold hyperalgesia [63]. TRPM subtype 8 (TRPM8) channels are also temperature-gated with the temperature threshold of 23–28 °C. In injury-induced neuropathic pain models, the expression levels of TRPV1 were upregulated in SNL and CCI but downregulated in SNI [56,64,65,66]. Due to their roles in neuropathic pain, TRP channels have become one of the therapeutic targets. For one, by inhibiting calmodlin-dependent protein kinass II (CAMKII) expression and extracellular signal regulated protein kinase 2 (ERK2) phosphorylation, researchers had successfully silenced the TRPV1 channels and thus attenuated neuropathic pain in rats [67]. Consequently, understanding the altered expression of TRP channels in various neuropathic pain models is essential in developing successful therapy.

#### 3.1.2. Voltage-Gated Sodium Channels (NaVs)

Typically, there are nine genes in the mammals that encode the different subunits of the voltage-gated sodium channels, NaV1.1–NaV1.9. These NaVs can be further categorized into two groups, tetrodotoxin-sensitive (TTX-S) and tetrodotoxin-resistant (TTX-R) based on their respective sensitivity to the neurotoxin tetrodotoxin (TTX). DRG neurons have the most diverse range of NaV subtypes compared to other neuronal cell types, up to five subtypes [68,69]. Matured DRG neurons have the capability to express both TTX-S (NaV1.1, NaV1.6, and NaV1.7) and TTX-R (NaV1.8 and NaV1.9) subtypes of NaVs. Among these NaVs, NaV1.7, NaV1.8, and NaV1.9 are specific subtypes in the neurons of the peripheral nervous system.

These NaVs have been linked with neuropathic pain. Several neuropathic pain models have shown to alter the expressions of NaVs at the transcription level i.e., the transcription can be “turned on” or “turned off” in an event of injury [70]. Increased expression of NaVs have been linked with painful diabetic neuropathy and injury-induced neuropathy [71,72,73]. Thus, the alternation in the expression of NaVs following neuropathic pain models is crucial in the understanding of neuropathic pain.

#### 3.1.3. Voltage-Gated Calcium Channels

Voltage-gated calcium channels are grouped into two classes: High-voltage activated type, the L, N, P/Q, and R type, and low-voltage activated type, also known as T-type [74]. All CaVs, except T-type CaVs, consist of three subunits [75], the α1 channel-forming subunit, the intracellular β subunit, and the α2δ subunit that consists of two disulfide-linked polypeptides (α2 and δ) encoded by the same gene [76]. These subunits may play an important role in neuropathic pain.

T-type CaVs, namely CaV3.2 and CaV3.3, are expressed in small and medium size isolectin B4 (IB4) (+) DRG neurons [77,78,79]. CaV3.2 plays an important role in antinociceptive, antihyperalgesic, and antiallodynic behavior after neuropathic pain of CCI [80], providing evidence that T-type CaVs can be potential therapeutic targets of neuropathic pain.

Both mRNA and protein levels of the α_2_δ-1 subunit of voltage-gated calcium channels were upregulated in SNL rats. Furthermore, the elevation of α_2_δ-1 was correlated with mechanical allodynia [81]. These results suggest that the α_2_δ-1 subunit could be a therapeutic target for peripheral neuropathic pain. However, in CCI-treated mice, several species of the mRNA that encode the α_1_ subunit of CaVs were downregulated [82]. The fluctuations of these subunits are considered to modulate the functions of CaVs. In the SNI model, T-type CaV3.2 receptors were upregulated [80]. The post-injury alteration in α_2_δ level, combined with the successful prevention of injury-induced pain by intrathecal infusion of gabapentin, provides convincing evidence for therapeutic possibilities of CaVs.

## 4. Epigenetic, Transcriptional, and Post-Translational Modification in Neuropathic Pain

In addition to the protein dogma theory, there are reports highlighting the participating epigenetic changes, translational modification, and post-translational control in various biological processes such as development, differentiation, proliferation, cell death, and cancer progression [83,84]. Several lines of evidence indicated that these regulations also participate in neuropathic pain [85,86,87]. Hence, personalize medicine could provide the optimized medical decision for different patients according to their personal genetic background such as single nucleotide polymorphism [88]. In this part, we will focus on several topics in peripheral neuropathic pain; (1) DNA methylation and acetylation, (2) the interactions between phosphorylated extracellular signal-regulated kinase (p-ERK) and transcriptional factors (TFs), and (3) ubiquitination and deubiquitination.

### 4.1. DNA Methyltransferase (DNAMT) and Histone Deacetylase (HDAC)

Epigenetic changes including DNA methylation, and histone acetylation regulate the gene expression without changing the coding sequence. The basic unit of chromatin, nucleosome, is composed of a segment of DNA and two copies of four histone proteins (H2A, H2B, H3, and H4) [89]. The folding pattern of chromatin DNA governs to the gene transcription efficiency. Chromatin enriched with methylation likely reduces the transcription efficiency. In contrast, the acetylated chromatin with less condensed DNA structures presents the most active transcriptional region [90]. Once the homeostasis of DNA methylation and acetylation is disrupted, the dysregulated gene expression will lead to various pathology or diseases such as cancer.

#### 4.1.1. DNA Methylation and Demethylation

Accumulated evidence has showed the potential contribution of DNA methylation and demethylation to the regulation of neuropathic pain behaviors [85,91]. DNA methylation was decreased in DRG after SNL and the hypomethylation leading to mechanical hypersensitivity by (1) intrathecal injection of DNA methyltransferase inhibitor, RG108 or (2) giving a methyl donor-deficient diet. The expression of genes related to voltage-gated and ligand-gated ion channels in DRG were identified and decreased based on the comparison between the RNA-seq data and the DNA methylation data [92]. Zhang et al. demonstrated that the demethylation on the promoter region of the P2X3 receptor was increased four weeks after injected a single dosage of streptozocin (STZ). The demethylated region of the P2X3 receptor could increase nuclear factor kappa B (NF-κB) binding to the P2X3 promoter followed with an increase in the expression level of P2X3 receptor [93]. The decreased voltage-dependent potassium channel subunit 2 (Kcna2) in DRG was observed in the neuropathic pain model of SNL and CCI. The expression of Kcna2 could be regulated by octamer transcription factor (OCT1) that binds to the promoter region of DNMT3a on day three post-SNL. The expression level of Kcna2 could be rescued after treating DNA methylation blocker [94].

#### 4.1.2. Histone Deacetylase Inhibitors

Histone deacetylase inhibitors are well developed and frequently used in cancer therapy. In recent years, HDACs have been implicated in the mechanisms of neuron degeneration and regulation of the neuronal plasticity [95,96,97]. For example, HDAC5 was increased in DRG on day seven after partial sciatic nerve ligation. The increased HDAC5 could bind to the Sox10 promoter region [98]. Matsushita et al. demonstrated that the activity of C-fiber could be reversed after treating HDAC inhibitors in partial sciatic nerve ligation-induced neuropathic pain model [99]. Mechanical and thermal hypersensitivities induced by ligation or transection of the spinal nerve were decreased after a treatment with class I HDAC inhibitors through the intrathecal route [100].

### 4.2. p-ERK and Transcriptional Factors

Mitogen-activated protein kinases (MAPKs) and TFs are molecules that responded rapidly to stress, hypoxia, and injury. Both MAPKs, especially ERK, and various TFs involve in peripheral neuropathic pain. p-ERK up-regulation in the dorsal horn neurons of the spinal cord contribute to the neuropathic pain of different nerve injuries [26,101,102]. The pain behaviors induced by chronic compression of the spinal nerve was highly correlated with an increase in phosphorylated-MAPKs including p-ERK, phosphorylated c-Jun N-terminal kinase (p-JNK), and phosphorylated p38 MAPK (p-p38)) in DRG at four days after surgery. The pain behaviors were reversed after treating MAPKs inhibitors [103]. Specific phosphorylation on the kinase insert domain in the nuclear translocation sequence, p-ERK interacts with a nuclear importing protein (importin-7) and then translocates from the cytosol into the nucleus [104]. Nuclear p-ERK regulates transcription factors and regulatory proteins to influence neuropathic pain mediators such as proto-oncogene c-fos and cAMP-response element binding protein (CREB) [105,106]. Upon peripheral nerve injury, these TFs were upregulated and translocated from the cytosol into the nucleus. The activated nuclear c-fos and CREB is regulated by p-ERK and then mediates the downstream gene involve in neuropathic pain [107].

p-ERK also regulates another transcription factor, activating transcription factors 3 (ATF3), a key mediator in nerve injury and regeneration [108,109]. Our previous study revealed that the increased co-localization of ATF3 and P2X3 on day seven after RTX treatment was highly correlated with resiniferatoxin induced neuropathic pain behaviors [59]. Ding et al. also demonstrated that ATF3 was increased three weeks after operation and participated in P2X3-induced endometriosis pain through ATF3/AP-1 activation [110]. Therefore, phosphorylation of ERK could be a potential therapeutic target for treating neuropathic pain in the future.

### 4.3. Ubiquitination and Deubiquitination

The ubiquitination and deubiquitination keep a delicate balance for post-translational modification system that facilitates the proteasomal pathway to regulate intracellular protein levels. Both ubiquitination and deubiquitination involve in neuropathic pain. E3 ubiquitin ligase regulates ion channel proteins [111,112,113]. For example, Nedd4-2 (neural precursor cell expressed, developmentally down-regulated 4-like, E3 ubiquitin protein ligase), an E3 ubiquitin ligase, is decreased on day 7 after SNI. The decreased Nedd4-2 is responsible for ubiquitination of NaVs, and caused dysregulation of NaV1.7 and NaV1.8 in the SNI model [73]. García–Caballero et al. (2014) showed that ubiquitin-specific protease 5 (USP5), a deubiquitinating enzyme, is increased on day 14 after CCI and modulates T-type CaV3.2 [114]. The depletion of USP5 in DRG successfully alleviated pain hypersensitivity in SNI mice, implicating the causal relationship between USP5 and pain induced by neuropathic models.

## 5. Current Challenges to Manipulate on DRG in In Vivo Models

DRGs which contain pseudounipolar neurons that perceive sensations from the terminal ending of nerve fiber then transduce back to the spinal cord [115]. After receiving the stimuli, DRG neurons will be activated by increasing the neuronal excitability and nociceptive signals [116]. Thus, to manipulate on DRG is a promising approach for investigating the molecular mechanisms of neuropathic pain. Previous studies revealed that direct injection into the DRG might trigger inflammatory response, while the intrathecal route might cause the injected component diffusible in multiple levels of the spinal cord [117,118,119]. We have established the spinal nerve injection technique to delivery gene constructs at a single site without removing the vertebrae and hence provided a feasible approach with a low risk of inflammation and injury [27]. There was no activation of ATF3 and ionized calcium binding adaptor molecule 1 (Iba1) in DRG neurons after spinal nerve injection in comparison with intra-DRG injection. Although the technique of spinal nerve injection presents much more challenge compared to intrathecal injection, spinal nerve injection is suitable for delivering drugs or gene construct into DRG in in vivo studies because some etiologies causing peripheral neuropathic pain is local but not systemic.

Gene therapy is a promising therapy for treating neuropathic pain. Several AAV clinical trials were already undergoing for Alzheimer’s disease, Parkinson’s disease, rheumatoid arthritis, etc. [120]. The serotype of AAV most widely used in clinical trial is AAV2 that work well in the CNS but with poor infection efficiency in DRG [121,122]. Several serotypes of AAV affect the infection efficiency, and the promoters on the construct also show huge variations on the ability of driving gene expression. Tissue specific promoters are designed and cloned into construct backbone to deliver gene expression to specific cell type in tissues [123]. Exposure route is another issue for concern in gene therapy. High titer of AAV virus particles must be used in systemic injection in which severe immune response might be triggered [124]. Because multiple systems or tissues are affected in many diseases, local injection the AAV virus particles might only be feasible in certain diseases such as gene mutation-related congenital blindness [125]. The above-mentioned issues might be considered carefully for selecting suitable AAV backbone, exposure rout, and serotype on clinical applications in the future.

## 6. Conclusions

The guidelines for treating neuropathic pain have been published by several international and regional professional associations. Antidepressants, anticonvulsants, and opioids are commonly used for treating neuropathic pain. However, these current therapy drugs like opioids do not directly target on pain-related molecules. The most challenges of the treatment on neuropathic pain are adverse effects, a lack of specificity, addiction, and inefficiency of pain relief. Although several voltage-gated sodium channel blockers were used to treat neuropathic pain, but the outcomes of these clinical trials still face the issue mentioned above [126]. Dexpramipexole, a specific blocker (NaV1.7 and NaV1.8) that effectively relieves neuropathic pain in various neuropathic pain models with well tolerance in several clinical trials, is undergoing clinical trial [127,128,129,130]. Therefore, targets on voltage-gated sodium channels could be the potential therapeutic targets in neuropathic pain. Due to the DRG is the first gate that connects the spinal cord and terminal nerve ending and receives the stimuli from nerve injury. Investigating the functions of pain-related genes and proteins in DRG through these different neuropathic pain models is necessary for screening potential targets. High throughput screening such as next generation sequencing, proteomics, and metabolomics are well-established approaches for searching novel candidate therapeutic targets. Combination of current therapeutic drugs with targeting novel molecules at the same time will reduce the side effects and be an alternative therapy in the future.

**Table 2:** Molecular mediators of neuropathic pain in focal neuropathy models.

DRG	P2Xs	TRPs	NaVs	CaVs
Injury-Induced				
SNL	P2X3 ↑ (IR: d7, mRNA: d1) [55,56,57] P2X3 ↓ (IR: d14, mRNA: d7) [55,57] P2X3—(IR: d3, mRNA: d14) [56] P2X4—(mRNA: d1, d7) [57] P2X5 ↑ (mRNA: d1, d7) [57] P2X6 ↓ (mRNA: d7) [57] P2X6—(mRNA: d1) [57]	TRPV1 ↑ (IR: d3, mRNA: d1-28) [56] TRPA1 ↑ (IR: d11, mRNA) [131]	NaV1.7 ↓ (L5, IR: d7, mRNA: d7) [132] NaV1.7 ↑ (L4, IR: d7, mRNA: d7) [132] NaV1.3 ↑ [133] NaV1.1 ↓ [133] NaV1.6 ↓ [133] NaV1.7 ↓ [133] NaV1.8 ↓ [133] NaV1.9 ↓ [133]	α_2_δ-1 subunit ↑ (mRNA: d2) [81]
CCI	P2X2—(IR: d18) [134] P2X2 ↑ (mRNA: d14) [135] P2X3 ↑ (IR: d14, d18, mRNA: d14) [51,52,53,58,135] P2X4—(IR: d18) [134] P2X4 ↓ (IR:d18, mRNA: d14) [51,53] P2X5—(mRNA: d14) [134] P2X6—(IR: d18) [134] P2X6 ↑ (IR: d18) [53]	TRPM8 ↑ (IR: d4, d7, d10, d14, mRNA: d14) [64,136] TPRMP—(mRNA: d7) [136] TRPA1 ↑ (mRNA: d7, d14, d16) [136,137] TRPV1 ↑ (mRNA: d16) [137] TRPV1—(mRNA: d7, d14) [136]	NaV1.7 ↑ (IR: d7, mRNA: d7) [132]	α 1 ↓ (mRNA: d7) [82]
SNI	P2X3—(IR: d7) [135]	TRPM8 ↓ [138] TRPV1 ↓ [138] TRPA1 ↓ [138]	Rats (protein) [139] NaV1.3 ↑ NaV1.7 ↓ NaV1.8 ↓ NaV1.9 ↓ Mice (mRNA) [140] NaV1.1 ↓ NaV1.2 − NaV1.3 ↓ NaV1.6 ↓ NaV1.7 ↓ NaV1.8 ↓ NaV1.9 ↓	Cav3.2 ↑ [80]

Abbreviation: IR, immunoreactivity; mRNA, messenger RNA. Notations: ↑, upregulation post-exposure of neuropathic pain. ↓, downregulation post-exposure of neuropathic pain. −, no significant change post-exposure of neuropathic pain. N/A, no direct evidence at the current time.

**Table 3:** Molecular mediators of neuropathic pain in systemic small fiber neuropathy models.

DRG	P2Xs	TRPs	NaVs	CaVs
Systemic Small Fiber Neuropathy				
RTX	P2X3 ↑ (IR: d7) [59]	TRPV1 ↓ (mRNA: d3) [53,59,136] TRPA1 ↓ (mRNA: d3) [136,141] TRPM8—(mRNA: d3) [136]	N/A	N/A
Cisplatin	P2X3 ↑ (mRNA: d14) [60]	TRPV1 ↑ [142] TRPA1 ↑ [142] TRPM8—[142]	N/A	CaV2.2 ↑ (IR: d3) [143]

Notations: ↑, upregulation post-exposure of neuropathic pain. ↓, downregulation post-exposure of neuropathic pain. —, no significant change post-exposure of neuropathic pain. N/A, no direct evidence at the current time.

## Figures and Tables

**Table 1:** Injury-induced neuropathic pain models.

	Advantage	Disadvantage	Application
SNL [21]	Specific injury in L5 and L6 DRG	Time consuming	Spinal nerve injection
CCI [22]	Combine injury and uninjured fibers	Variation in each operation	Decompression model
SNI [23]	Combine injury and uninjured fibers Robustness of the response	Low local inflammation	

Abbreviation: SNL, spinal nerve ligation; CCI, chronic constriction injury; and SNI, spared nerve injury.
